# Predicting the Initiation of Continuous Venovenous Hemofiltration in Acute Pancreatitis Patients: The Role of Plasma and Urinary Neutrophil Gelatinase-Associated Lipocalin

**DOI:** 10.3390/jcm15072509

**Published:** 2026-03-25

**Authors:** Le Huu Nhuong, Le Viet Thang, Nguyen Trung Kien, Pham Thai Dung, Nguyen Quoc Khanh, Hoang Thuy, Nguyen Van Tam, Pham Dang Thuan

**Affiliations:** 1 Military Hospital 354, Hanoi 100000, Vietnam; lehuunhuong@gmail.com (L.H.N.); khanhnguyenquoc354@gmail.com (N.Q.K.); 2 Military Hospital 103, Hanoi 100000, Vietnam; dzungdoctor@gmail.com (P.T.D.); bstampttk@gmail.com (N.V.T.); 3 Department of Nephrology and Hemodialysis, Vietnam Military Medical University, Hanoi 100000, Vietnam; drkien103@gmail.com; 4 Military Medical Department, General Department of Logistics and Technical Services, Ministry of National Defense, Hanoi 100000, Vietnam; 5 National Lung Hospital, Hanoi 100000, Vietnam; hthuybs@gmail.com; 6 Department of Emergency and Critical Care Medicine, Thai Binh University of Medicine and Pharmacy, Thai Binh 410000, Vietnam; phamthuan.ytb@gmail.com

**Keywords:** acute pancreatitis, acute kidney injury, NGAL, continuous venovenous hemofiltration, renal replacement therapy

## Abstract

**Background**: Acute kidney injury (AKI) is a serious complication of acute pancreatitis and is frequently associated with the need for continuous renal replacement therapy. Early identification of patients at risk of requiring continuous venovenous hemofiltration (CVVHF) remains challenging because conventional renal markers often reflect delayed kidney injury. Neutrophil gelatinase-associated lipocalin (NGAL) has emerged as a potential biomarker of early renal tubular damage. **Methods**: This observational study included 219 patients with acute pancreatitis. Plasma and urinary NGAL levels were measured at hospital admission. Clinical characteristics, laboratory parameters, and severity scores were compared between patients who required CVVHF and those who did not. Multivariate logistic regression analysis was performed to identify factors associated with CVVHF requirement, and predictive performance was evaluated using receiver operating characteristic (ROC) curve analysis. **Results**: During hospitalization, 28 patients (12.8%) required CVVHF and had significantly more severe disease. Both plasma and urinary NGAL levels were higher in patients requiring CVVHF. In multivariate analysis, urinary NGAL remained independently associated with CVVHF requirement. ROC analysis demonstrated moderate predictive performance for urinary NGAL (AUC 0.708). **Conclusions**: Urinary NGAL was independently associated with the requirement for CVVHF and demonstrated moderate predictive performance. These findings suggest that urinary NGAL may provide kidney-specific information and improve early risk stratification beyond conventional clinical parameters.

## 1. Introduction

Acute pancreatitis (AP) is a disease spectrum ranging from mild to severe with an unpredictable natural course. The annual incidence of AP is estimated at 13–49 per 100,000 persons. The majority of cases (approximately 80%) are mild and self-limiting. However, severe AP (SAP) has a mortality risk of up to 30% [1]. The risk of AP is similar among men and women and increases with age [2]. Despite advances in supportive care, severe acute pancreatitis continues to impose a substantial burden on intensive care resources worldwide [3]. Among systemic complications, acute kidney injury (AKI) is particularly devastating, occurring in approximately 20–40% of patients with severe AP and markedly increasing the risk of mortality [4]. The pathophysiology of AKI in acute pancreatitis is complex and multifactorial. Systemic inflammatory response and hypovolemia are primary drivers of renal injury. Notably, microcirculatory impairment plays a crucial role and is closely associated with renal vasoconstriction and endothelial dysfunction, which together contribute to renal hypoperfusion and tubular injury [5]. When AKI progresses or is accompanied by refractory metabolic disturbances, continuous renal replacement therapy (CRRT), particularly continuous venovenous hemofiltration (CVVHF), is frequently required. Importantly, the need for CVVHF in AP has consistently been associated with poor outcomes and high mortality, reflecting severe multiorgan dysfunction rather than isolated renal failure [6,7].

Early identification of patients who will require CVVHF remains a major clinical challenge. Conventional renal markers, such as serum creatinine and urine output, are limited by delayed elevation and poor sensitivity for early tubular injury, especially in critically ill patients with fluid shifts and altered muscle mass. Similarly, widely used clinical severity scores—including APACHE II, SOFA, BISAP, and Marshall—provide valuable global risk stratification but lack kidney-specific sensitivity and may fail to detect early renal injury before overt deterioration occurs [8,9].

In recent years, there has been growing interest in biomarkers that directly reflect renal tubular damage. Neutrophil gelatinase-associated lipocalin (NGAL) is a small glycoprotein released by injured tubular epithelial cells and activated neutrophils in response to ischemic and inflammatory insults [10]. Both plasma and urinary NGAL levels rise rapidly following kidney injury, preceding increases in serum creatinine by several hours to days. Accumulating evidence from critically ill and heterogeneous ICU populations suggests that NGAL may predict severe AKI and the need for renal replacement therapy earlier than conventional markers [11,12]. In the context of acute pancreatitis, several recent studies have suggested that elevated NGAL levels are associated with disease severity, persistent organ failure, and AKI [13,14,15]. However, data specifically evaluating the role of plasma and urinary NGAL in predicting the requirement for CVVHF in acute pancreatitis remain limited and inconsistent. Moreover, most existing studies have focused on AKI as an endpoint rather than the clinically critical decision to initiate continuous renal replacement therapy.

Although several studies have investigated NGAL in acute pancreatitis, most have focused on the prediction of AKI rather than clinically meaningful endpoints such as renal replacement therapy. Moreover, comparative data between plasma and urinary NGAL remain limited. A preliminary analysis of this cohort has been previously reported in a research letter focusing on the comparative performance of urinary and plasma NGAL for predicting CVVHF requirement [16]. However, that report was limited in scope and did not include comprehensive multivariable modeling or integration with established clinical severity scores. Therefore, the present study aimed to provide a more detailed and clinically oriented evaluation of the predictive value of NGAL by incorporating clinical, biochemical, and radiological parameters to better define its role in early risk stratification.

## 2. Materials and Methods

### 2.1. Study Design and Population

This observational study was conducted at Bach Mai Hospital and Military Hospital 354, Hanoi, Vietnam, between December 2021 and December 2023. Adult patients (≥18 years) admitted with a diagnosis of acute pancreatitis were consecutively screened for eligibility.

The diagnosis of acute pancreatitis was established according to the revised Atlanta classification (2012), requiring at least two of the following three criteria: (1) typical abdominal pain consistent with acute pancreatitis; (2) serum amylase or lipase levels at least three times the upper limit of normal; (3) imaging findings characteristic of acute pancreatitis on abdominal ultrasound, computed tomography (CT), or magnetic resonance imaging (MRI) [17].

Patients were categorized into two groups: AKI group: patients with acute pancreatitis complicated by acute kidney injury; Non-AKI group: patients with acute pancreatitis without acute kidney injury.

Acute kidney injury (AKI) was diagnosed and staged according to the Kidney Disease: Improving Global Outcomes (KDIGO) 2012 criteria, defined as an increase in serum creatinine ≥26.5 μmol/L within 48 h or ≥1.5 times baseline [18].

Additionally, a reference group of healthy individuals matched for age and sex was included to determine baseline plasma and urinary NGAL levels.

#### 2.1.1. Inclusion and Exclusion Criteria

Inclusion criteria: Patients were eligible if they met the following criteria:

- Age ≥18 years;
- Diagnosis of acute pancreatitis according to the Atlanta 2012 criteria;
- Provided informed consent to participate in the study.

Exclusion criteria: Patients were excluded if they had: pre-existing chronic kidney disease; history of renal transplantation; malignancy; acute pancreatitis associated with systemic poisoning or advanced multiorgan failure; death within 48 h of hospital admission; insufficient blood or urine samples for NGAL measurement. Healthy individuals were excluded if they had: acute infections; history of renal disease; chronic pancreatitis.

#### 2.1.2. Sample Size

The sample size was calculated using the formula for estimating a single proportion:

$$n=Z^{2}\times \frac{p\times (1-p)}{e^{2}}$$

where

*Z* = 1.96 (95% confidence level)

*p* = 7.9%, the reported incidence of AKI in acute pancreatitis according to Devani et al. [19].

*d* = 0.08 (acceptable margin of error)

The minimum required sample size was 44 patients with AKI. In the present study, 51 patients with AKI were included, satisfying the sample size requirement.

#### 2.1.3. Clinical and Laboratory Data Collection

Baseline demographic and clinical data were recorded at admission, including: Age; Sex; Body mass index (BMI); Comorbidities; Clinical symptoms of acute pancreatitis; Vital signs recorded included: Heart rate; Blood pressure; Respiratory rate; Oxygen saturation; Body temperature; Routine laboratory tests were performed within the first 24 h of admission.

Hematology tests: Complete blood count was performed using an automated hematology analyzer (Sysmex XN-1000, Kobe, Japan) at the central laboratory of Bach Mai Hospital.

Measured parameters included: Red blood cells; Hemoglobin; Hematocrit; White blood cells; Platelet count;

Biochemical analysis: Serum biochemical parameters included: Glucose; Urea; Creatinine; AST; ALT; Bilirubin; Albumin; Lactate dehydrogenase (LDH); Triglycerides; Electrolytes (Na^+^, K^+^, Cl^−^, Ca^2+^); C-reactive protein (CRP). All biochemical analyses were performed using automated biochemical analyzers (Cobas 8000, Roche Diagnostics, Basel, Switzerland) that meet ISO 15189 laboratory standards [20].

Arterial blood gas analysis: Arterial blood gases were measured to determine: pH; pO_2_; pCO_2_; HCO_3_^−^; Lactate; PaO_2_/FiO_2_ ratio.

#### 2.1.4. Imaging Assessment

All patients underwent abdominal ultrasound and contrast-enhanced CT scans to evaluate pancreatic inflammation and complications.

The severity of acute pancreatitis was assessed using: Balthazar classification; Computed Tomography Severity Index (CTSI).

Clinical severity scores: Disease severity was assessed using established scoring systems: APACHE II; SOFA; BISAP; Imrie (Glasgow) score; Modified Marshall score.

Severe acute pancreatitis was defined according to the revised Atlanta classification (2012) based on persistent organ failure.

##### Measurement of Plasma and Urinary NGAL

Plasma and urinary NGAL concentrations were measured using a sandwich enzyme-linked immunosorbent assay (ELISA).

Blood samples (3 mL) were collected in EDTA tubes and centrifuged at 2000 rpm for 10 min. Plasma was separated and stored at −80 °C until analysis.

Urine samples were collected in sterile tubes, centrifuged under the same conditions, and stored at −20 °C until measurement.

NGAL levels were quantified using a commercial ELISA kit (Sigma-Aldrich, St. Louis, MO, USA) according to the manufacturer’s instructions.

Optical density was measured at 450 nm using an ELISA microplate reader (DAR 800, Diagnostic Automation Inc., Calabasas, CA, USA). NGAL concentrations were determined based on a standard calibration curve.

Outcome measures: The primary outcome of this study was the requirement for continuous venovenous hemofiltration (CVVHF) during hospitalization. Secondary outcomes included the occurrence and severity of acute kidney injury (AKI), as defined by KDIGO criteria, and in-hospital mortality.

The decision to initiate CVVHF was made by experienced intensivists and nephrologists based on standard clinical indications, including hemodynamic instability, refractory metabolic acidosis, severe electrolyte imbalance, fluid overload, and progressive AKI. In critically ill patients with acute pancreatitis, continuous renal replacement therapy (CRRT), particularly CVVHF, is generally preferred over intermittent hemodialysis due to better hemodynamic tolerance and more precise fluid management.

#### 2.1.5. Statistical Analysis

Statistical analyses were performed using SPSS version 26.0 (IBM Corp., Armonk, NY, USA).

Continuous variables were expressed as mean ± standard deviation or median with interquartile range, depending on distribution.

Comparisons between groups were performed using: Student’s *t*-test; Mann–Whitney U test; Chi-square test or Fisher’s exact test.

Correlations between NGAL levels and clinical variables were assessed using Pearson or Spearman correlation analysis.

The predictive value of plasma and urinary NGAL was evaluated using receiver operating characteristic (ROC) curve analysis.

Diagnostic performance was assessed by calculating: Area under the curve (AUC); sensitivity; specificity. The optimal cutoff value was determined using the Youden index.

A *p*-value < 0.05 was considered statistically significant.

#### 2.1.6. Ethical Approval

The study was approved by the Ethics Committee of Bach Mai Hospital (Approval No. 3094/BVBM-HĐĐĐ).

All participants or their legal representatives provided informed consent prior to inclusion in the study.

## 3. Results

The baseline clinical characteristics and laboratory parameters are presented in Table 1. Multivariate logistic regression analysis of factors associated with CVVHF requirement is shown in Table 2. The predictive performance of plasma and urinary NGAL is summarized in Table 3.

**Figure 1:**
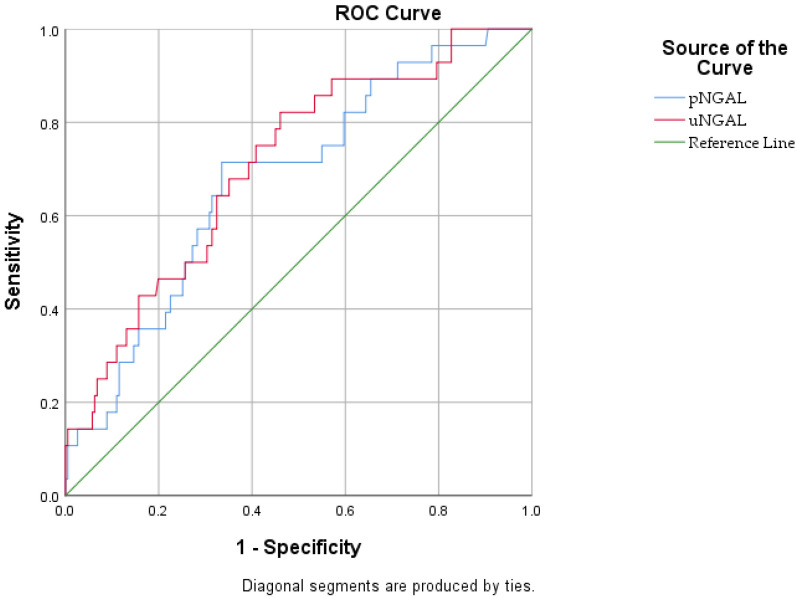
Receiver operating characteristic (ROC) curves of plasma and urinary NGAL for prediction of continuous venovenous hemofiltration in patients with acute pancreatitis.

The predictive performance of plasma and urinary NGAL for CVVHF requirement was evaluated using receiver operating characteristic (ROC) curve analysis (Figure 1). Overall, these findings indicate that urinary NGAL provides superior and independent predictive value for CVVHF requirement compared with plasma NGAL, supporting its role as a kidney-specific biomarker in this clinical setting.

OR: odds ratio, CI: confidence interval, NGAL: neutrophil gelatinase-associated lipocalin, APACHE II: Acute Physiology and Chronic Health Evaluation II, BISAP: Bedside Index for Severity in Acute Pancreatitis, CRP: C-reactive protein

## 4. Discussion

In this observational study, we demonstrated that the requirement for continuous venovenous hemofiltration (CVVHF) represents a clinically meaningful endpoint reflecting severe acute kidney injury and multiorgan dysfunction in patients with acute pancreatitis. By integrating clinical severity scores, conventional laboratory parameters, kidney-specific biomarkers, and predictive performance analysis, our findings provide a comprehensive nephrology-focused framework for early identification of patients at high risk for CVVHF.

Patients requiring CVVHF exhibited significantly more severe acute pancreatitis, characterized by higher rates of pancreatic necrosis, more advanced Balthazar grades, and higher CTSI scores. These findings are consistent with previous studies showing that extensive pancreatic necrosis and severe radiological involvement are strongly associated with persistent organ failure and the need for renal replacement therapy in acute pancreatitis [21]. Severe pancreatic injury triggers a sustained systemic inflammatory response, endothelial dysfunction, and microcirculatory impairment, which together contribute to multiorgan failure, including acute kidney injury (AKI) [22]. Recent multicenter studies have also demonstrated that patients with Balthazar grade D–E and high CTSI scores are at particularly high risk of developing AKI and subsequently requiring continuous renal replacement therapy (CRRT) [23,24]. Clinical severity scores, including APACHE II, SOFA, IMRIE, BISAP, and Marshall scores, were significantly higher in patients requiring CVVHF, underscoring the role of global disease severity and multiorgan dysfunction. Consistent with prior reports, APACHE II and SOFA showed the strongest associations with adverse outcomes and RRT requirement (Table 1) [6,25]. However, these composite scores rely on multiple physiological variables and may not adequately capture early kidney-specific injury. BISAP, although useful for early risk stratification, did not retain independent predictive value for CVVHF after adjustment, in line with studies suggesting limited sensitivity for evolving organ dysfunction beyond the early phase of disease [26,27].

AKI was markedly more prevalent in the CVVHF group and was accompanied by significantly elevated serum urea and creatinine levels. The pathophysiology of AKI in this context is multifactorial, involving hypovolemia, systemic inflammation, and notably, microcirculatory impairment, which is closely associated with renal vasoconstriction and endothelial dysfunction. These factors collectively lead to direct tubular injury. Importantly, serum creatinine is a delayed marker of renal functional change and often fails to reflect early structural tubular damage, particularly in critically ill patients with fluid shifts [28].

Metabolic and electrolyte disturbances were more pronounced in patients requiring CVVHF, particularly hypocalcemia and hyperkalemia. Hypocalcemia has been consistently associated with severe acute pancreatitis, pancreatic necrosis, persistent organ failure, and AKI, reflecting fat saponification, cytokine-mediated calcium redistribution, and impaired parathyroid hormone response [29]. Recent studies have identified low serum calcium as an independent predictor of RRT and mortality in severe acute pancreatitis [29]. Hyperkalemia, reflecting impaired renal excretion and metabolic acidosis, represents a classical indication for CVVHF and further emphasizes the severity of renal dysfunction in this subgroup [30].

Arterial blood gas analysis revealed significant acidosis, elevated lactate levels, and impaired oxygenation in patients requiring CVVHF. These abnormalities indicate tissue hypoperfusion and mitochondrial dysfunction and respiratory compromise, all of which contribute to AKI progression and the need for RRT. Prior studies have demonstrated that early metabolic acidosis and reduced PaO_2_/FiO_2_ ratios independently predict persistent organ failure and CRRT initiation in acute pancreatitis. Moreover, metabolic acidosis and hyperlactatemia are direct clinical triggers for initiation of CVVHF in critically ill patients [31].

Importantly, our findings highlight the differential clinical significance of plasma and urinary NGAL. While both biomarkers were elevated in patients requiring CVVHF, only urinary NGAL retained independent predictive value after adjustment. This is biologically plausible, as urinary NGAL directly reflects renal tubular epithelial injury, whereas plasma NGAL may be influenced by systemic inflammation and extrarenal sources. Therefore, urinary NGAL provides more kidney-specific and clinically relevant information for predicting the need for renal replacement therapy. These findings are consistent with accumulating evidence that urinary NGAL more accurately reflects renal tubular injury, whereas plasma NGAL may be confounded by systemic inflammation and extrarenal sources in critical illness (Table 2) [32]. Recent studies in acute pancreatitis and heterogeneous ICU populations have shown that urinary NGAL independently predicts severe AKI and RRT, even after adjustment for APACHE II or SOFA scores. These data support the role of urinary NGAL as a kidney-specific biomarker that provides prognostic information beyond global severity indices [32].

ROC curve analysis further supported the clinical utility of NGAL in predicting the need and initiation of CVVHFF. Urinary NGAL demonstrated moderate discriminative ability, with an area under the curve (AUC) of 0.708 at an optimal cutoff value of 7.5 ng/mL. Notably, this cutoff achieved a high sensitivity of 82.1% at the early stage of admission, which is crucial for identifying the ‘therapeutic window’ where clinicians can anticipate the progression toward renal replacement therapy before overt clinical deterioration occurs (Table 3).

We observed that our optimal cutoff for urinary NGAL (7.5 ng/mL) is relatively lower than those reported in some previous studies involving general ICU populations. This discrepancy may be attributed to several factors, including the early timing of our measurements (within 24 h of admission) and the specific pathophysiology of acute pancreatitis. Unlike septic shock or post-cardiac surgery AKI, where systemic insults may be more abrupt, the early phase of acute pancreatitis involves a unique cytokine-mediated inflammatory profile that may trigger lower but significant early releases of NGAL from stressed tubular cells. These findings underscore the importance of using disease-specific and time-sensitive cutoffs to optimize the early risk stratification of patients in this clinical setting.

Although established clinical severity scores generally demonstrate stronger global predictive performance, urinary NGAL achieved comparable sensitivity despite being a single kidney-specific biomarker measured early during hospitalization. Previous studies have suggested that combining urinary NGAL with clinical severity scores improves the prediction of renal replacement therapy compared with either approach alone [33]. From a nephrology perspective, this integrated strategy may enhance early risk stratification and support timely clinical decision-making regarding renal support.

This study has several strengths, including a well-characterized cohort, a comprehensive assessment of disease severity, integration of kidney-specific biomarkers, and robust statistical analysis. However, several limitations should be acknowledged. First, the observational design precludes causal inference. Second, NGAL was measured at a single time point, and serial measurements may provide additional prognostic insight. Third, the single-country design may limit generalizability, although it contributes valuable data from an underrepresented population.

Compared with our previous preliminary report, the present study provides a more comprehensive evaluation by incorporating a broader range of clinical variables and validated severity scores. Importantly, we demonstrate that urinary NGAL retains independent predictive value even after adjustment for established prognostic indices, supporting its incremental clinical utility.

Importantly, our findings suggest that urinary NGAL may serve as a practical and early biomarker to guide clinical decision-making in critically ill patients with acute pancreatitis. By identifying patients at high risk of requiring CVVHF at an early stage, clinicians may optimize hemodynamic management and initiate timely renal support, potentially improving outcomes.

This study has several limitations. First, the relatively small number of patients requiring CVVHF may limit the robustness of multivariate analyses and increase the risk of overfitting. Second, NGAL was measured at a single time point, and serial measurements may provide additional prognostic value. Third, the single-country design may limit generalizability. Therefore, larger multicenter studies are warranted to validate these findings.

## 5. Conclusions

In patients with acute pancreatitis, the need for continuous venovenous hemofiltration reflects severe disease with acute kidney injury and multiorgan dysfunction. Urinary NGAL was independently associated with the requirement for CVVHF and showed moderate predictive performance. Incorporating urinary NGAL into early clinical assessment may help improve risk stratification and support timely decisions regarding renal replacement therapy.

## Figures and Tables

**Table 1:** Comparison of clinical characteristics and laboratory parameters of patients with CVVHF (+) and CVVHF (−).

Characteristics	All (*n* = 219)	CVVHF (+), (*n* = 28)	CVVHF (−), (*n* = 191)	*p*
Age, years	45.84 ± 13.14	46.64 ± 13.97	45.72 ± 13.05	0.73
Gender, *n* (%)				
‒ Male	180 (82.2)	27 (96.4)	153 (80.1)	0.034
‒ Female	39 (17.8)	1 (3.6)	38 (19.9)	
BMI (kg/m^2^)				
‒ <18.5	17 (7.8)	2 (7.1)	15 (7.8)	0.263
‒ 18.5–22.9	87 (39.7)	7 (25.0)	80 (41.9)	
‒ 23–<25	58 (26.5)	8 (28.6)	50 (26.2)	
‒ ≥25	57 (26.0)	11 (39.3)	46 (24.1)	
‒ Mean	23.29 ± 4.37	23.61 ± 3.63	23.25 ± 4.47	0.689
Blood pressure, mmHg				
‒ Systolic	130.11 ± 20.98	122.00 ± 25.16	131.29 ± 20.11	0.028
‒ Diastolic	79.03 ± 13.85	75.21 ± 16.74	79.59 ± 13.34	0.119
‒ Mean	96.07 ± 14.95	90.83 ± 18.47	96.84 ± 14.26	0.047
Comorbidities, *n*(%)				
‒ Hypertension	34 (15.5)	5 (17.9)	29 (15.2)	0.78
‒ Diabetes	30 (13.7)	8 (28.6)	22 (11.5)	0.033
Etiology, *n*(%)				
‒ Lipid disorders	48 (21.9)	6 (21.4)	42 (22.0)	0.58
‒ Gallstone	10 (4.6)	0 (0)	10 (5.2)	0.247
‒ Alcohol	95 (43.4)	17 (60.7)	78 (40.8)	0.038
Acute pancreatitis				
‒ First time	125 (57.1)	20 (71.4)	105 (55.0)	0.36
‒ Once	56 (25.6)	4 (14.3)	52 (27.2)	
‒ Twin	14 (6.4)	2 (7.1)	12 (6.3)	
‒ ≥3 times	24 (11.0)	2 (7.1)	22 (11.5)	
Hematology				
‒ Leucocyte	12.83 ± 3.90	10.34 ± 3.59	13.11 ± 3.03	0.82
‒ Hemoglobin	137.45 ± 27.97	166.00 ± 24.04	134.28 ± 27.09	0.61
‒ Platelet	217 ± 45	154.50 ± 48.79	224.44 ± 67.37	0.73
Blood metabolism				
‒ Glucose	10.13 ± 5.61	10.76 ± 10.84	10.06 ± 5.31	0.003
‒ Protein	64.28 ± 8.19	62.75 ± 3.04	64.45 ± 8.61	<0.001
‒ Albumin	34.45 ± 5.82	36.75 ± 4.87	34.20 ± 5.98	0.001
‒ LDH	441.00 ± 435.29	671.50 ± 369.81	415.39 ± 443.60	<0.001
‒ AST	46.32 ± 54.15	49.00 ± 4.10	46.03 ± 57.23	0.131
‒ ALT	44.54 ± 56.52	22.80 ± 3.39	46.95 ± 59.23	0.465
‒ Billirubin	29.11 ± 34.81	27.05 ± 3.04	29.34 ± 39.79	0.240
‒ Amylase	635.91 ± 850.26	736.65 ± 485.57	624.71 ± 890.39	0.637
‒ Lipase	968.93 ± 1754.8	2334.50 ± 1723.22	817.20 ± 1738.73	0.975
‒ TG	15.52 ± 26.05	8.82 ± 1.66	16.26 ± 27.43	0.452
‒ CRP	20.68 ± 26.34	25.71 ± 10.53	20.12 ± 27.67	0.438
Arterial blood gas				
‒ pH	7.42 ± 0.06	7.34 ± 0.07	7.43 ± 0.05	<0.001
‒ pCO_2_	33.90 ± 4.79	37.50 ± 10.61	33.51 ± 4.17	0.092
‒ pO_2_	85.75 ± 25.04	71.50 ± 71.42	87.34 ± 19.35	0.278
‒ HCO_3_^−^	22.34 ± 3.71	19.45 ± 6.72	22.66 ±3.42	<0.001
‒ P/F ratio	395.83 ± 136.11	341.95 ± 342.17	401.82 ± 115.93	0.033
‒ Lactate	2.5 ± 2.31	6.78 ± 5.53	2.03 ± 1.32	<0.001
‒ Prothrombin (s)	13.42 ± 1.75	10.85 ± 1.20	13.7 ± 1.57	<0.001
‒ INR	1.12 ± 0.14	0.99 ± 0.12	1.14 ± 0.13	<0.001
AKI				
‒ Urea	5.01 ±2.89	7.8 ± 3.96	4.71 ± 2.72	<0.001
‒ Creatinine	77.70 ± 45.75	169.50 ± 30.41	67.50 ± 34.40	<0.001
‒ AKI	51 (23.3)	21 (75.0)	30 (15.7)	<0.001
‒ Na^+^	128.86 ± 30.41	136.50 ± 3.53	128.01 ± 32.02	0.975
‒ K^+^	3.66 ± 0.53	3.80 ± 1.11	3.64 ± 0.48	0.003
‒ Ca	2.04 ± 0.36	1.76 ± 0.67	2.06 ± 0.33	<0.001
‒ Cl^−^	98.44 ± 6.35	95.70 ± 1.84	98.75 ± 6.63	0.477
Acute pancreatitis				
‒ Mild, moderate	160 (73.1)	5 (17.9)	155 (81.2)	<0.001
‒ Severe	59 (26.9)	23 (82.1)	36 (18.8)	
‒ Oedema	159 (73.3)	16 (57.1)	143 (75.7)	0.064
‒ Necrosis	58 (26.7)	12 (42.9)	46 (24.3)	
‒ Balthazar B + C + D	89 (40.6)	4 (14.3)	85 (44.5)	0.002
‒ Balthazar E	130 (59.4)	24 (85.7)	106 (55.5)	
‒ CTSI	4.21 ± 1.79	5.22 ± 2.24	4.06 ± 1.68	0.002
APACHE II	6.15 ± 4.36	13.00 ± 1.41	5.39 ± 3.86	<0.001
SOFA	1.9 ± 1.94	5.00 ± 2.83	1.56 ± 1.58	<0.001
IMRIE	2.05 ± 1.64	2.50 ± 2.12	2.00 ± 1.64	<0.001
BISAP	1.10 ± 1.07	2.00 ± 1.41	1.00 ± 1.03	<0.001
MARSHALL	1.00 ± 1.34	3.50 ± 2.12	0.72 ± 0.96	<0.001
Plasma NGAL	661.63 ± 732.11	2627.39 ± 839.29	443.21 ±229.09	<0.001
Urine NGAL	264.83	1127.98 ± 1300.82	168.92 ± 130.91	<0.001
Mortality	3 (1.4)	2 (7.1)	1 (0.5)	0.044

BMI body mass index, SOFA Sequential Organ Failure Assessment, APACHE II Acute Physiology and Chronic Health Evaluation II, PF ratio PaO_2_/FiO_2_ ratio, NGAL neutrophil gelatinase-associated lipocalin, p-plasma, u-urine, BISAP Bedside Index of Severity in Acute Pancreatitis, CTSI Computed Tomography Severity Index, AKI acute kidney injury, data are presented as mean ± standard deviation or N (%). Note: Urinary NGAL was analyzed for all 219 patients. In cases of oliguria, samples were obtained via urinary catheterization.

**Table 2:** Multivariate logistic regression analysis between CVVHF and some clinical variables in patients with acute pancreatitis.

	Adjusted OR	95% CI	*p*
uNGAL	0.997	0.995–1.000	0.016
pNGAL	0.999	0.998–1.001	0.371
APACHE II	0.748	0.593–0.944	0.014
BISAP	1.395	0.634–3.067	0.408
Total Calcium	11.475	1.559–84.454	0.017
CRP	0.999	0.996–1.003	0.677
Lactat	0.901	0.571–1.424	0.656

Patients requiring CVVH had significantly more severe disease, as reflected by higher APACHE II, SOFA, BISAP, and Marshall scores. These patients also demonstrated more frequent pancreatic necrosis and higher CTSI scores, indicating more extensive pancreatic injury. Renal dysfunction was markedly more pronounced in the CVVH group, with significantly elevated serum urea and creatinine levels and a higher prevalence of AKI.

**Table 3:** Predictive performance of plasma and urinary NGAL for continuous venovenous hemofiltration.

Variable(s)	AUC (95% CI)	Cut-Off (ng/mL)	*p*	Sensitivity (%)	Specificity (%)
Plasma NGAL	0.678 (0.609–0.835)	201.96	0.002	75.00	75.24
Urinary NGAL	0.708 (0.631–0.834)	7.5	0.000	82.1	71.65

## Data Availability

The original contributions presented in this study are included in the article. Further inquiries can be directed to the corresponding author.
